# Sound touch viscosity in the evaluation of median nerve in healthy volunteers—a preliminary study

**DOI:** 10.1186/s12880-025-01939-z

**Published:** 2025-09-29

**Authors:** Jun Huang, Hongpeng Duan, Minwei Zhang, Jian Lu, Feng Mao, Ling Zhou, Shengmin Zhang

**Affiliations:** Department of Medical Ultrasound, The First Affiliated Hospital of Ningbo University, No.59 LiuTing Street, Ningbo, 315010 China

**Keywords:** Viscosity, Ultrasound, Shear wave elastography, Median nerve, Healthy volunteers

## Abstract

**Background:**

Quantitative assessment of peripheral nerve viscoelasticity is important for understanding nerve physiology and detecting early neuropathic changes. However, reference values for the viscosity and stiffness of the median nerve in healthy adults are lacking.

**Materials and methods:**

A total of 98 healthy volunteers (58 females, 40 males; mean age: 35 ± 12 years) were assessed using the Sound Touch Viscosity and shear wave elastography modules integrated into the Resona A20S ultrasound system. Viscosity and stiffness were measured at three anatomical locations: the carpal tunnel (MN1), mid-forearm (MN2), and 5 cm proximal to the elbow joint (MN3). Paired bilateral measurements were obtained, and demographic factors were analyzed for their influence.

**Results:**

No significant differences were found between the left and right median nerves (*p* > 0.05). MN1 showed the highest viscosity 1.83 Pa·s (1.48–2.13) and stiffness of 30.24 kPa (28.46–33.65) significantly greater than MN2 and MN3 (*p* < 0.001). Viscosity and stiffness were moderately correlated at all sites ((*r* = 0.39, 0.56, and 0.36; all *p* < 0.001). Males showed higher stiffness at all locations (*p* < 0.001) and higher viscosity at MN1 and MN2 (*p* = 0.039 and 0.011). While age and body mass index (BMI) showed no significant effects.

**Conclusion:**

STVi is a feasible and reproducible modality for quantifying median nerve viscoelasticity. Viscoelastic parameters vary significantly by anatomical location and sex, with no influence from age and BMI. These findings establish normative values and support the clinical applicability of Sound Touch Viscosity in peripheral nerve assessment.

**Supplementary Information:**

The online version contains supplementary material available at 10.1186/s12880-025-01939-z.

## Introduction

Over the past few decades, ultrasound technology has made significant strides in evaluating the biomechanical properties of biological tissues. Shear wave elastography (SWE), a well-established noninvasive and quantitative technique, has been extensively applied in clinical practice to assess tissue stiffness [1–4]. However, conventional SWE operates under the assumption that tissues are homogeneous, purely elastic, and isotropic, thereby overlooking the influence of viscous components [5]. Specifically, it assumes that shear wave propagation velocity is independent of shear wave frequency—a simplification that fails to capture the complex mechanical behavior of biological tissues.

In reality, biological tissues are nonlinear, heterogeneous, and viscoelastic rather than purely elastic [6, 7]. While elasticity primarily determines the propagation speed of shear waves, viscosity contributes to wave dispersion, a phenomenon wherein shear wave velocity varies with frequency [6, 8]. These dispersion effects can be quantified by analyzing the nonlinear relationship between the applied acoustic radiation force and the resulting tissue displacement [8–10]. Therefore, elasticity measurements alone may yield biased estimates [11], whereas a combined viscoelastic analysis offers a more comprehensive depiction of the underlying pathophysiological state, particularly in peripheral nerve.

The recent development of the Sound Touch Viscosity (STVi) module has addressed the gap in quantitative viscosity assessment. For example, the Mindray Resona A20S ultrasound system, equipped with a high-frequency linear transducer (LM18-5WU), enables real-time visualization and quantification of tissue viscosity in peripheral nerves by applying a viscoelastic fitting model [12]. Viscosity values are acquired at specific anatomical locations and expressed in Pascal seconds (Pa·s), thereby providing localized mechanical insight.

Despite this technical progress, two major knowledge gaps remain. First, the feasibility and reliability of STVi for peripheral nerve evaluation have not yet been systematically examined in terms of repeatability and reproducibility. Second, normative reference values for median nerve (MN) viscosity and stiffness in healthy adults are lacking, and the potential influence of demographic factors such as age, sex, and body mass index (BMI) on these parameters remains unclear. Although several studies have investigated stiffness of peripheral nerves using SWE, quantitative viscosity data are almost absent, and whether demographic characteristics introduce physiological variability is still unknown.

We selected the median nerve as the target of investigation because it is the most clinically relevant peripheral nerve, being frequently affected in common neuropathies such as carpal tunnel syndrome. Establishing normative viscoelastic values in healthy volunteers therefore not only provides a technical reference but also creates a foundation for identifying pathological deviations in future patient cohorts. Moreover, STVi may complement existing diagnostic tools, such as conventional SWE and electromyography (EMG), by simultaneously quantifying both elasticity and viscosity, thereby offering a more comprehensive characterization of nerve mechanics.

Accordingly, the primary aim of this study is to establish baseline normative values for MN viscosity and stiffness in healthy volunteers, thereby providing a technical foundation for future clinical applications. The secondary aim is to explore the associations of demographic factors with MN viscoelasticity, which may help to elucidate sources of biological variability and guide clinical interpretation. These dual objectives not only clarify the feasibility of STVi as a novel imaging modality but also highlight its potential clinical significance for early detection and monitoring of peripheral neuropathies.

## Materials and methods

### Study population

This prospective study was conducted between August and October 2024 in the Department of Ultrasound at a tertiary academic hospital. A total of 113 healthy adult volunteers were randomly recruited from the hospital community. Individuals were eligible for inclusion if they were aged 18 years or older and able to cooperate with ultrasound procedures. Exclusion criteria included pregnancy; a history or clinical suspicion of peripheral neuropathy; systemic conditions known to affect peripheral nerve function, such as diabetes mellitus, hypothyroidism, chronic alcohol use, vitamin B12 deficiency, or chronic kidney disease; and localized inflammation, trauma, surgical scarring, or skin lesions at the anatomical measurement sites.

To ensure the absence of both overt and subclinical median nerve pathology, a standardized three-step screening protocol was implemented prior to enrollment.

- Functional evaluations were performed by rehabilitation physicians to assess motor and sensory function according to the Clinical Guidelines for Rehabilitation Medicine. Participants were required to exhibit normal muscle strength (≥ grade 5 on the Medical Research Council scale) and intact sensation on standard clinical examination.
- Physical examinations were conducted by hand surgeons, including Tinel’s sign and Phalen’s test, following the Diagnostic Standards for Upper Limb Peripheral Neuropathies. A negative result on both tests was required for inclusion.
- Structural assessments were performed using high-resolution B-mode ultrasonography by an experienced musculoskeletal sonographer with at least five years of experience in peripheral nerve imaging. The median nerve was required to demonstrate a normal fascicular echotexture, with no evidence of swelling, loss of fascicular pattern, or other sonographic abnormalities.

Abnormal findings were defined as the presence of any of the following: reduced muscle strength, sensory deficit on examination, positive Tinel’s sign and Phalen’s test, or sonographic abnormalities (abnormal course, uneven thickness, disordered sieve-like reticular structure, heterogeneous echogenicity, or epineurial thickening). Only participants who exhibited no abnormal findings in all three evaluations were deemed eligible.

**Fig. 1:**
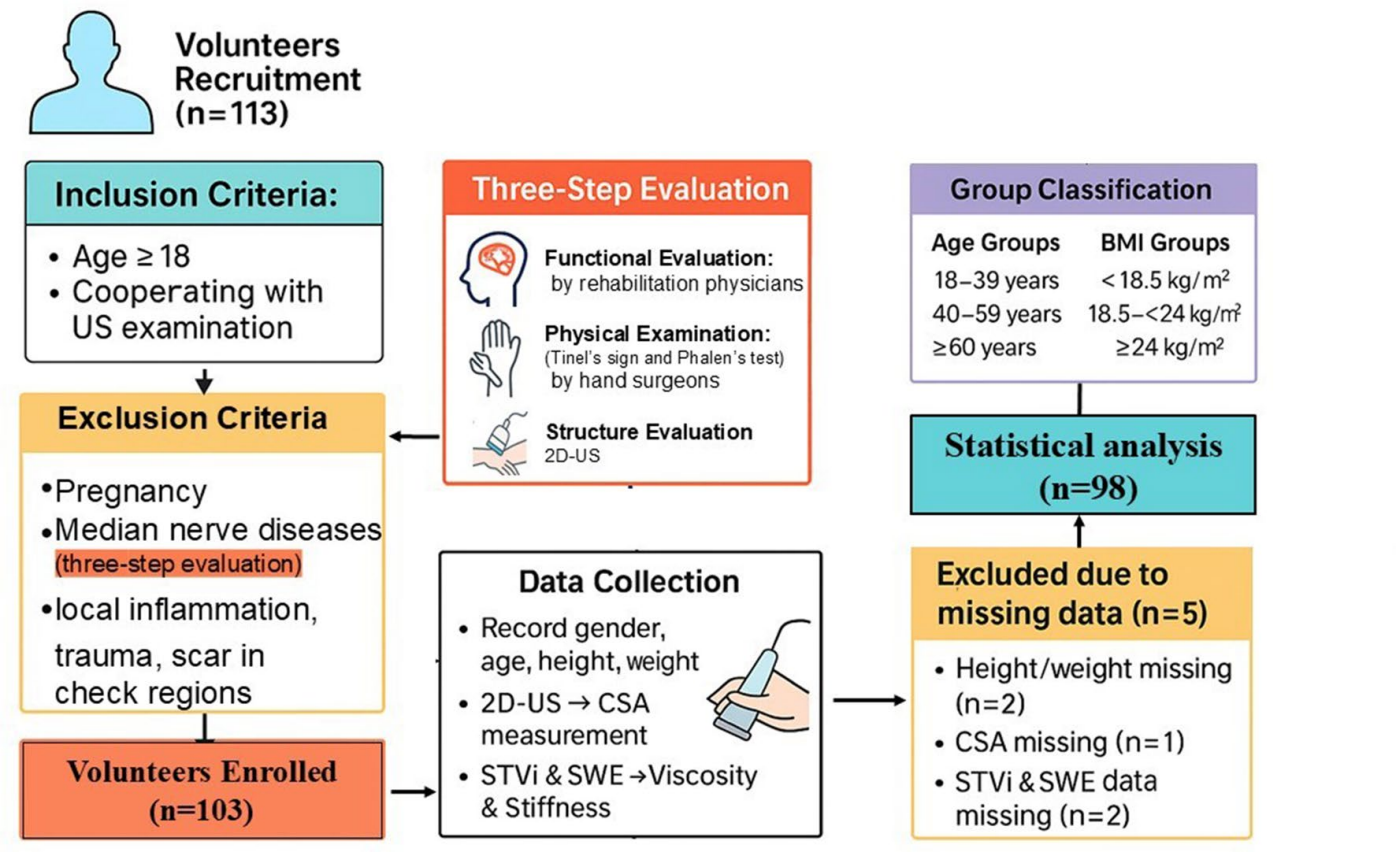
Flowchart of participant recruitment, screening, and data collection. A total of 113 volunteers were recruited. Following exclusion and multi-step screening (clinical, physical, and ultrasound evaluation), 103 were enrolled and completed measurements. Data from 98 participants were included in the final analysis

Of the 113 individuals initially recruited, 15 were excluded from the final analysis due to the following reasons: two had missing height and weight data, one lacked cross-sectional area (CSA) measurements, two had incomplete STVi or SWE values, five were excluded due to abnormal findings during screening, and five voluntarily withdrew before completing the protocol. Ultimately, 98 healthy adults were included in the final dataset. The study cohort comprised 58 females (59.2%) and 40 males (40.8%), with a mean age of 35 ± 12 years and an average BMI of 22.60 ± 3.20 kg/m². For each participant, demographic characteristics including age, sex, height, and weight were recorded, and BMI was calculated accordingly (Fig. 1).

Participants were stratified into three age groups (18–39 years, 40–59 years, and ≥ 60 years) and three BMI categories (underweight, < 18.5 kg/m²; normal weight, 18.5–23.9 kg/m²; and overweight/obese, ≥ 24.0 kg/m²) for subgroup analysis. The study protocol was approved by the institutional ethics committee (IRB No. 017 A/2023), and all procedures adhered to the principles of the Declaration of Helsinki (revised 2000, Edinburgh). Written informed consent was obtained from all participants prior to enrollment.

### Viscosity and stiffness measurements

Viscosity and stiffness values of the median nerve were assessed using the STVi module integrated into the Resona A20S ultrasound system (Shenzhen Mindray Biomedical Electronics Co., Ltd., Shenzhen, China), equipped with a high-frequency linear array transducer (LM18-5WU; center frequency ~ 12 MHz, bandwidth 5–18 MHz). The STVi mode evaluates shear wave dispersion by measuring propagation speeds across multiple frequencies, thereby providing simultaneous quantification of both viscosity and stiffness [13].

Technical implementation details:

- Shear wave frequencies between 200 and 800 Hz were selected for viscosity quantification.
- Shear wave propagation was tracked using plane-wave imaging acquired by the same probe at ~ 10,000 Hz frame rate, ensuring sufficient temporal resolution.
- The reliability (RLB) map was generated by integrating signal-to-noise ratio, shear wave propagation stability, and tissue motion consistency in the imaging plane.
- The acoustic transmit sequence (number and spacing of push pulses, focus depth, repetition rate) follows proprietary algorithms of the manufacturer and cannot be disclosed due to confidentiality restrictions.

All measurements were performed by a board-certified attending physician with more than 5 years of experience in peripheral nerve ultrasound, who was blinded to participants’ clinical data. To minimize operator-related bias, all measurements were conducted according to a standardized protocol following dedicated training sessions.

Ultrasound examinations were conducted in a quiet, temperature-controlled room (21–25 °C). Participants were positioned supine with upper limbs relaxed alongside the body. Bilateral median nerves were evaluated at three anatomical locations:

1. MN1: carpal tunnel level, defined as the midpoint between the scaphoid and pisiform bones;
2. MN2: mid-forearm, defined as the midpoint between the radial head and radial styloid, corresponding approximately to the junction of the proximal and middle thirds of the forearm. In transverse view, the median nerve at this level is typically located between the flexor digitorum superficialis and flexor digitorum profundus muscles;
3. MN3: 5 cm proximal to the elbow joint, with the medial epicondyle of the humerus serving as a reproducible bony landmark.

**Fig. 2:**
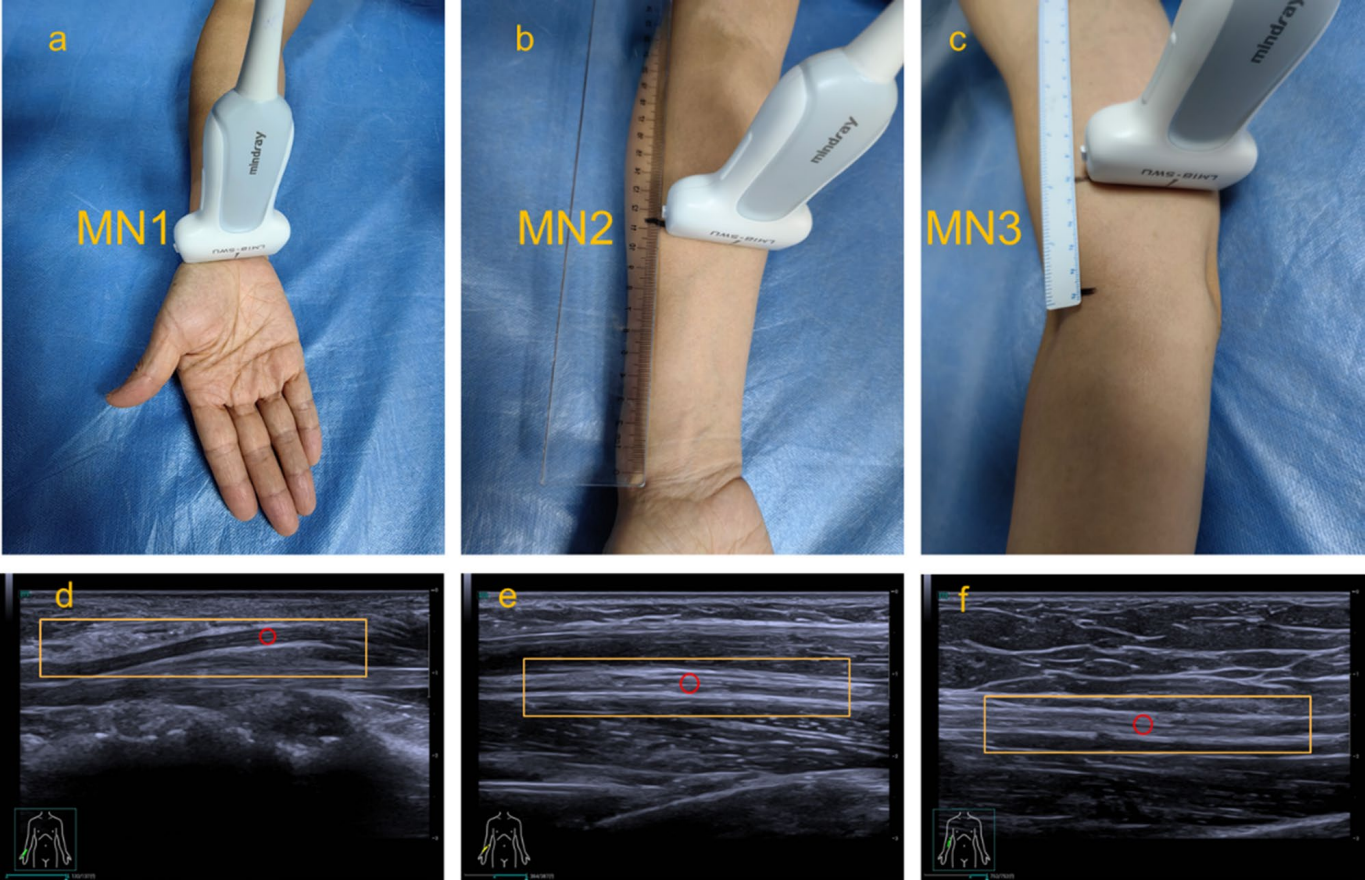
Representative probe placement and corresponding STVi measurements of the MN at three anatomical sites in a healthy volunteer. (**a**–**c**) Probe positioning for MN1, MN2, and MN3, respectively. (**d**–**f**) Corresponding longitudinal grayscale ultrasound images with the region of interest (ROI) indicated by the rectangular box and circular Q-box placed within the nerve for viscosity and stiffness quantification

**Fig. 3:**
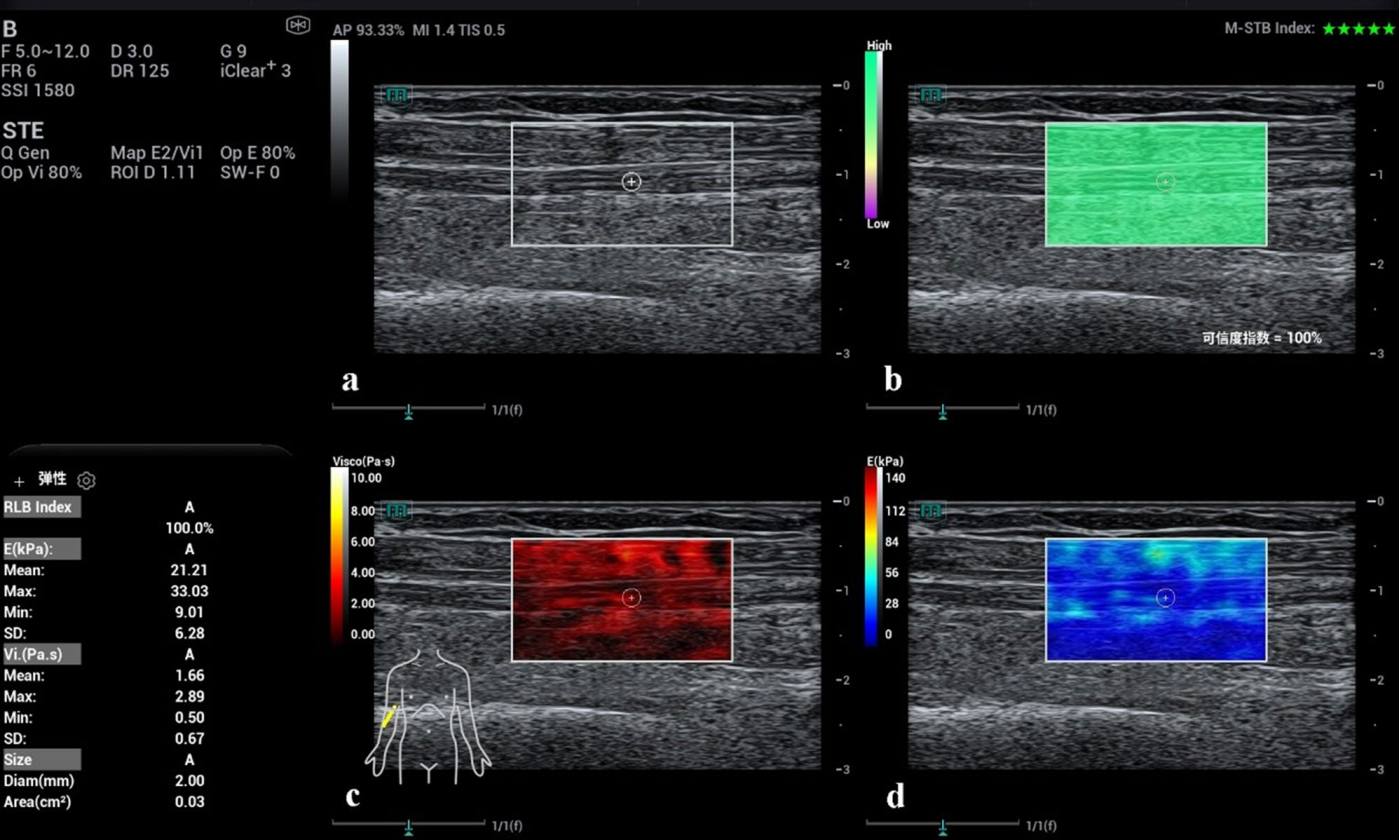
Representative STVi measurement of the right MN3 in a healthy subject. (**a**) Grayscale ultrasound image; (**b**) Reliability (RLB) map indicating high data quality (green); (**c**) Viscosity map showing localized distribution (range 0–10 Pa·s); (**d**) SWE map displaying stiffness distribution (range 0–140 kPa). Mean viscosity and stiffness values are displayed in the lower-left corner

The scanning depth was uniformly set to 2–3 cm for MN1–MN3. Representative B-mode images for each site, with ROI (Q-box) placement indicating the median nerve, are shown in Fig. 2. During scanning, the probe was held vertically with minimal pressure, and a generous amount of bubble-free coupling gel was applied. CSA was measured in the transverse view, followed by rotation of the transducer to obtain longitudinal views for viscoelastic imaging. Once a clear acoustic window was obtained, the viscoelastic mode was activated, and data acquisition began only when the motion stability index (M-STB index) displayed 4 or 5 green stars, indicating optimal image stability. A quad-view layout was used to simultaneously display grayscale, RLB, viscosity, and SWE maps (Fig. 3):

- The grayscale map outlined tissue morphology;
- The RLB map indicated data reliability (green: high, purple: low), with valid measurements requiring ≥ 90% reliability;
- The viscosity map displayed viscosity distribution (range: 0–10 Pa·s), where red represented lower values and yellow/white higher values;
- The SWE map visualized stiffness distribution (range: 0–140 kPa), with blue indicating soft and red indicating stiff regions.

Each anatomical site was measured three times by the same operator, and the mean of these three values was used for analysis to ensure intra-operator repeatability. To further assess inter-operator reproducibility, 20 randomly selected participants were re-examined by two additional independent operators following the same acquisition protocol.

### Statistical analysis

Statistical analyses were performed using MedCalc Version 20 (MedCalc Software Corp., Brunswick, ME, USA) and GraphPad Prism 8.0.1. Normality of data distribution was assessed using the Shapiro–Wilk test. Continuous variables with normal distribution were expressed as mean ± standard deviation (SD), while non-normally distributed data were reported as median (Q25-Q75). Paired-sample t-tests or Wilcoxon signed-rank tests were used to compare differences between left and right median nerves. Differences between sex groups were analyzed using independent-sample t-tests or Mann–Whitney U tests, as appropriate. For comparisons across age and BMI categories, one-way analysis of variance (ANOVA) or Kruskal–Wallis H tests were employed, depending on data distribution. Pearson’s correlation coefficients were calculated for normally distributed variables to assess linear relationships. For non-normally distributed variables, Spearman’s rank correlation (rho) was used. A two-tailed *p*-value < 0.05 was considered statistically significant in all tests.

To evaluate inter-rater reliability, the intraclass correlation coefficient (ICC) was calculated using SPSS software (IBM SPSS Statistics, version 27.0). A two-way mixed-effects model with the consistency definition was applied, and both single-measure and average-measure ICC values with 95% confidence intervals were reported. In addition, ICCs were also computed for the subset of 20 participants re-examined independently by three operators (inter-operator reproducibility). Statistical power for the primary outcome comparisons was assessed post hoc, based on the total sample size (*n* = 98), an α level of 0.05, and the observed effect sizes.

## Results

### Participant characteristics

A total of 98 healthy adult volunteers were included in the final analysis, comprising 58 females (59.2%) and 40 males (40.8%). The mean age was 35 ± 12 years, and the average BMI was 22.60 ± 3.20 kg/m². Detailed demographic characteristics are summarized in Table 1.

**Table 1:** Demographic characteristics of the study group

Descriptor	*N* (%)
Volunteers	98
Sex	
Female	58 (59.2)
Male	40 (40.8)
Age (years)	
18–39	77 (78.6)
40–59	14 (14.3)
≥60	7 (7.1)
BMI (kg/m²)	
Underweight (< 18.5)	7 (7.1)
Normal (18.5–<24)	58 (59.2)
Overweight (≥ 24)	33 (33.7)

Values are presented as number of participants with percentages in parentheses [N (%)]

### Symmetry between bilateral median nerves

Paired comparisons revealed no statistically significant differences in either viscosity or stiffness between the left and right median nerves across all anatomical locations (all *p* > 0.05; Table 2). Median values and interquartile ranges were nearly identical between sides, further supporting the anatomical and biomechanical symmetry of the median nerve in healthy individuals. On this basis, and to avoid potential statistical non-independence, data from the dominant side were selected as the representative values for all subsequent analyses. The reliability of repeated measurements was further confirmed by intra- and inter-operator ICC analyses, which are reported in Supplementary Table S1 and S2.

**Table 2:** Comparisons of stiffness and viscosity values of bilateral MN in the same volunteer. (*n* = 98)

Parameter	MN1			MN2			MN3		
	Right	Left	*p*	Right	Left	*p*	Right	Left	*p*
CSA (mm²)	8 (7–9)	8 (7–9)	0.503^a^	8.5 (8–9)	8 (7–9)	0.290^a^	12 (11–14)	13 (11–14)	0.051^a^
Stiffness (kPa)	30.24 (28.46–33.65)	29.94 (28.37–32.93)	0.074^a^	21.58 (19.99–24.26)	21.75 (20.34–24.65)	0.187^a^	21.59 (19.65–23.45)	21.79 (20.05–23.82)	0.189^a^
Viscosity (Pa·s)	1.82 ± 0.63	1.89 ± 0.62	0.313^b^	1.58 (1.16–1.86)	1.60 (1.18–1.94)	0.782^a^	1.36 (1.13–1.69)	1.39 (1.10–1.72)	0.895^a^

Abbreviations: MN1, median nerve at the carpal tunnel; MN2, median nerve at mid-forearm; MN3, median nerve at 5 cm proximal to the elbow joint; CSA, cross-sectional area; Pa·s, pascal seconds; ^a^ Wilcoxon signed-rank tests, Median (Q25-Q75); ^b^ paired-sample t-tests

### Regional variation of viscoelastic properties

**Fig. 4:**
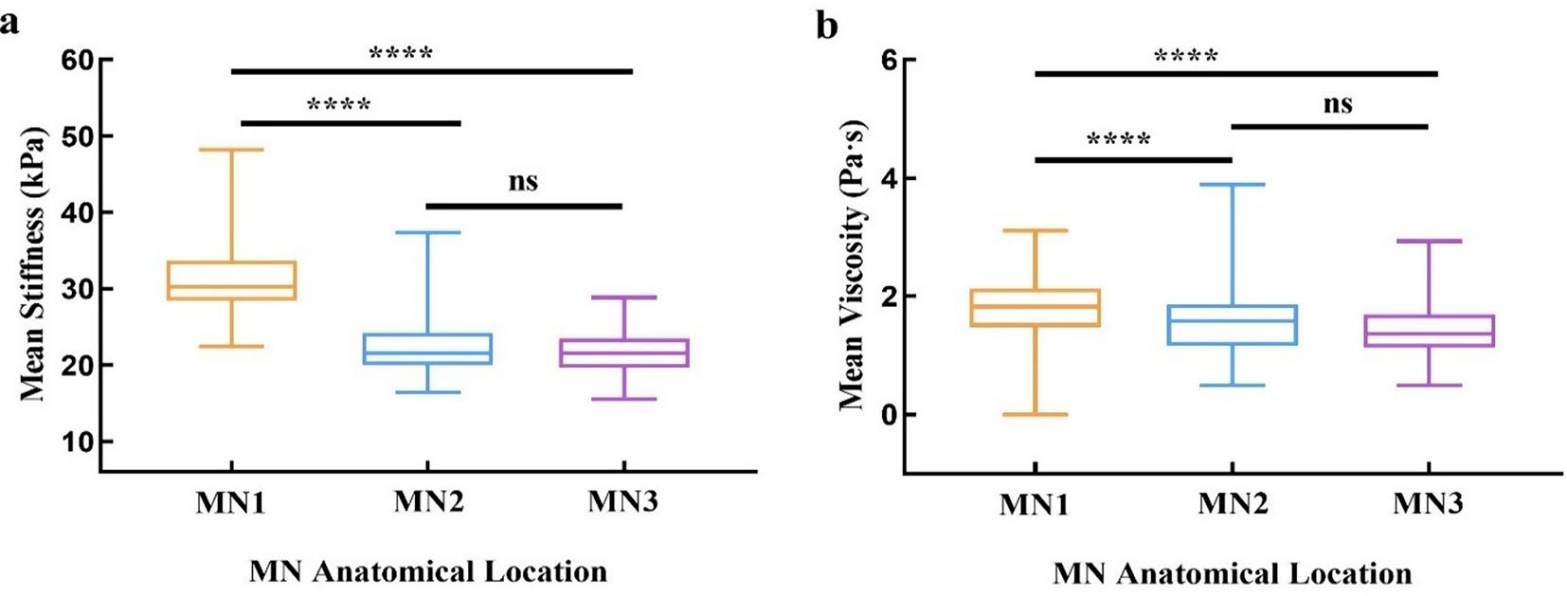
Anatomical location-specific differences in median nerve stiffness and viscosity. (**a**) Stiffness values significantly differed among MN1, MN2, and MN3; (**b**) Viscosity values also showed significant site-dependent variation. Kruskal–Wallis H test used; **** *p* < 0.001, * *p* < 0.05, ns: not significant

Both viscosity and stiffness differed significantly across the three anatomical sites (*p* < 0.001 for both; Table 3; Fig. 4). MN1 showed the highest values [viscosity: 1.83 Pa·s (1.48–2.13); stiffness: 30.24 kPa (28.46–33.65)], exceeding those at MN2 [1.58 Pa·s (1.16–1.86); 21.58 kPa (19.99–24.26)] and MN3 [1.36 Pa·s (1.13–1.69); 21.59 kPa (19.65–23.45)]. All pairwise comparisons were significant (*p* < 0.001), except for viscosity between MN2 and MN3 (*p* = 0.168) and stiffness between MN2 and MN3 (*p* > 0.999).

**Table 3:** Comparisons of stiffness and viscosity values of different sites of the dominant-side median nerve in the volunteers (*n* = 98)

Parameter	MN1	MN2	MN3	*p*-value	MN1 vs. MN2	MN1 vs. MN3	MN2 vs. MN3
CSA (mm²)	8 (7–9)	8.5 (8–9)	12 (11–14)	< 0.001^a^	> 0.999	< 0.001	< 0.001
Stiffness (kPa)	30.24 (28.46–33.65)	21.58 (19.99–24.26)	21.59 (19.65–23.45)	< 0.001^a^	< 0.001	< 0.001	> 0.999
Viscosity (Pa·s)	1.83 (1.48–2.13)	1.58 (1.16–1.86)	1.36 (1.13–1.69)	< 0.001^a^	0.009	< 0.001	0.168
Correlation							
S* and CSA	*p* = 0.065	*p* = 0.120	*p* = 0.703	- Not applicable- - - - - - -			
Vi* and CSA	*p* = 0.350	*p* = 0.833	*p* = 0.987				
Vi* and S*	*p* < 0.001 *r* = 0.39	*p* < 0.001 *r* = 0.56	*p* < 0.001 *r* = 0.36				

Abbreviations: MN1, median nerve at the carpal tunnel; MN2, median nerve at mid-forearm; MN3, median nerve at 5 cm proximal to the elbow joint; CSA, cross-sectional area; Pa·s, pascal seconds; S*, stiffness; Vi*, viscosity; ^a^ Kruskal–Wallis H test. Median (Q25-Q75)

**Fig. 5:**
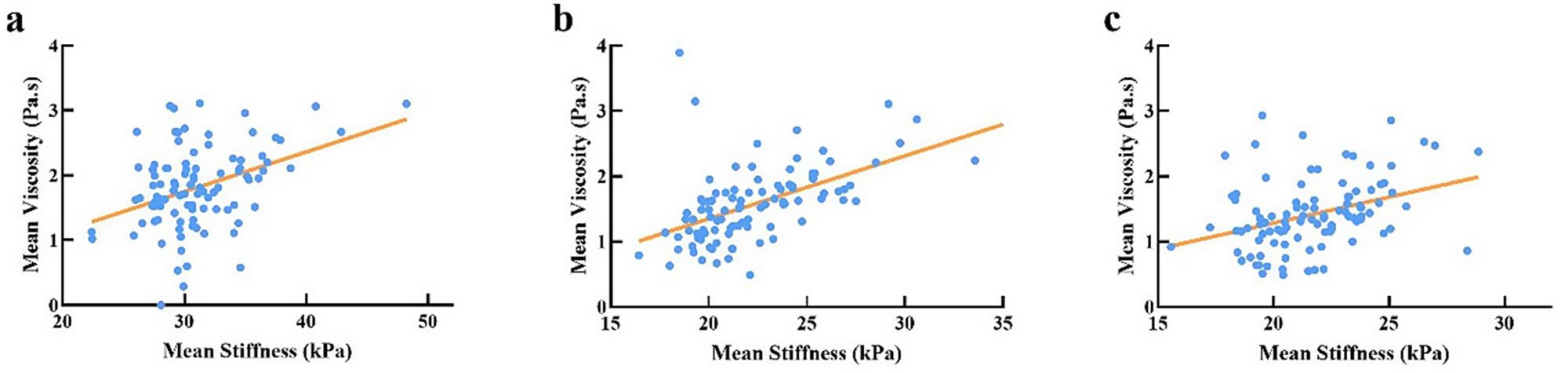
Correlation between viscosity and stiffness at three anatomical locations. Scatter plots show significant positive correlations between viscosity and stiffness at (**a**) MN1, (**b**) MN2, and (**c**) MN3. Pearson correlation coefficients provided; all *p* < 0.001

A moderate positive correlation was observed between viscosity and stiffness across all anatomical sites (*r* = 0.39, 0.56, and 0.36; all *p* < 0.001; Fig. 5). By contrast, no significant correlations were detected between CSA and either stiffness or viscosity at any of the three locations (all *p* > 0.05; Table 3).

### Sex-Based differences in median nerve viscoelasticity

**Fig. 6:**
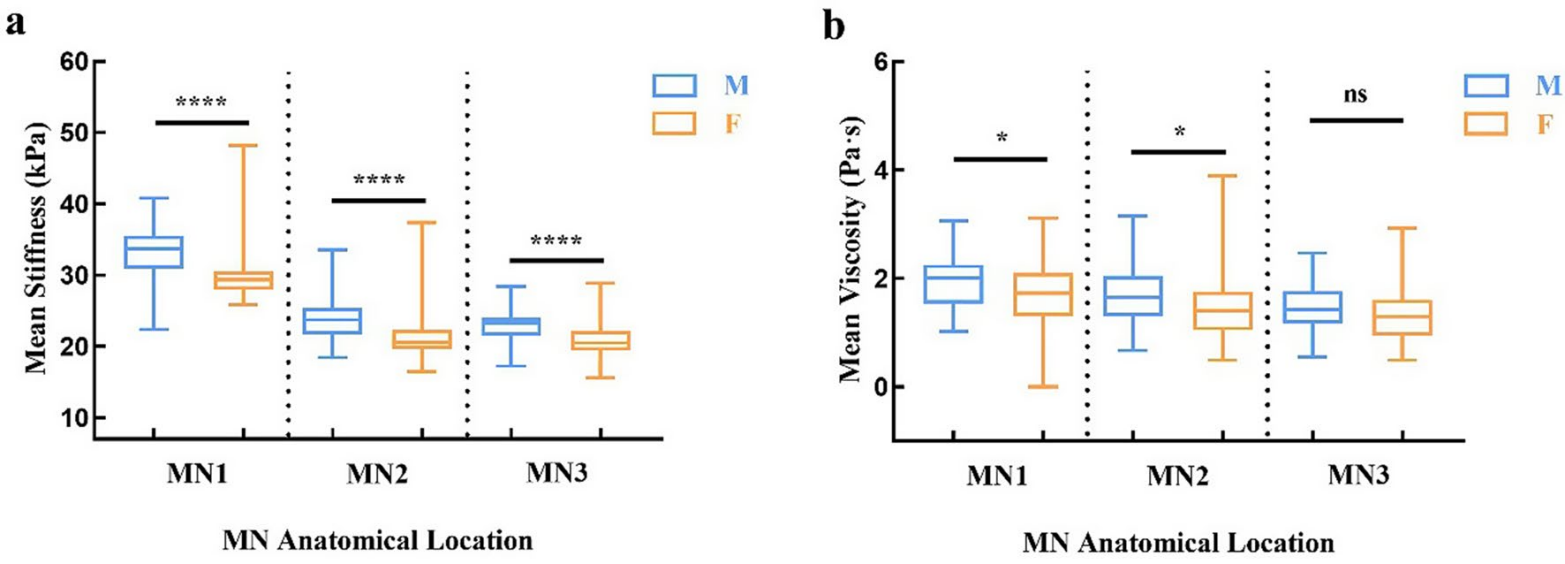
Sex-related differences in viscoelastic parameters of the median nerve. (**a**) Stiffness values were higher in males than females at all sites (**** *p* < 0.001); (**b**) Viscosity was significantly higher in males at MN1 (* *p* = 0.039) and MN2 (* *p* = 0.011). Mann–Whitney U or independent-sample t tests were used as appropriate

Male participants exhibited significantly higher stiffness values than females at all three anatomical locations (MN1–MN3, all *p* < 0.001; Table 4; Fig. 6). Specifically, stiffness at MN1 was 33.76 kPa (30.94–35.52) in males versus 29.35 kPa (27.92–30.53) in females (*p* < 0.001). At MN2, stiffness was 23.73 kPa (21.62–25.38) in males compared with 20.54 kPa (19.61–22.28) in females (*p* < 0.001). At MN3, stiffness values were 23.23 kPa (21.52–24.07) in males and 20.48 kPa (19.43–22.18) in females (*p* < 0.001).

**Table 4:** Comparisons of stiffness and viscosity values of the MN between male and female volunteers on the dominant side (*n* = 98)

Parameter	Site	Male (*n* = 40)	Female (*n* = 58)	*p*-value
CSA (mm²)	MN1	9 (8–10)	8 (7–9)	0.001^a^
	MN2	9 (8–10)	8 (7–9)	0.001^a^
	MN3	14 (12–15)	11 (10–13)	< 0.001^a^
Stiffness (kPa)	MN1	33.76 (30.94–35.52)	29.35 (27.92–30.53)	< 0.001^a^
	MN2	23.73 (21.62–25.38)	20.54 (19.61–22.28)	< 0.001^a^
	MN3	23.23 (21.52–24.07)	20.48 (19.43–22.18)	< 0.001^a^
Viscosity (Pa·s)	MN1	1.94 ± 0.53	1.73 ± 0.68	0.039^b^
	MN2	1.65 (1.30–2.05)	1.41 (1.05–1.75)	0.011^a^
	MN3	1.42 (1.17–1.77)	1.30 (0.95–1.61)	0.174^a^

Abbreviations: MN1, median nerve at the carpal tunnel; MN2, median nerve at mid-forearm; MN3, median nerve at 5 cm proximal to the elbow joint; CSA, cross-sectional area; Pa·s, pascal seconds; ^a^ Wilcoxon signed-rank tests, Median (Q25-Q75); ^b^ independent-sample t-tests

For viscosity, sex-related differences were also evident at MN1 and MN2. At MN1, males showed higher viscosity (1.94 ± 0.53 Pa·s vs. 1.73 ± 0.68 Pa·s, *p* = 0.039), consistent with the independent-sample t test used for normally distributed data. At MN2, viscosity values were significantly greater in males (1.65 Pa·s (1.30–2.05)) compared with females (1.41 Pa·s (1.05–1.75), *p* = 0.011), based on the Mann–Whitney U test. In contrast, no statistically significant difference was detected at MN3 (1.42 Pa·s (1.17–1.77) in males vs. 1.30 Pa·s (0.95–1.61) in females, *p* = 0.174).

Post-hoc power analyses confirmed that the cohort had adequate statistical power (> 0.80) to detect medium-to-large effect sizes, except for viscosity at MN3 where the effect size was small and power was limited (see Supplementary Table S3).

### Associations of age and BMI with median nerve viscoelasticity

Age-related comparisons revealed no statistically significant differences in either stiffness or viscosity across the three age groups (18–39, 40–59, ≥ 60 years) at any anatomical site (all *p* > 0.05; Table 5). For example, stiffness at MN1 was 30.28 kPa (28.84–34.02) in participants aged 18–39 years, 30.70 kPa (28.63–33.18) in those aged 40–59 years, and 27.81 kPa (27.43–31.08) in those aged ≥ 60 years (*p* = 0.319). Similarly, viscosity at MN1 was 1.84 ± 0.61 Pa·s, 1.90 ± 0.77 Pa·s, and 1.39 ± 0.43 Pa·s across the same groups, respectively (*p* = 0.168). Comparable nonsignificant results were observed at MN2 and MN3.

**Table 5:** Comparisons of stiffness and viscosity values of the MN on the dominant side in volunteers based on age distribution (*n* = 98)

Parameter	Site	G1 (18–39 y, *n* = 77)	G2 (40–59 y, *n* = 14)	G3 (≥ 60 y, *n* = 7)	*p*-value
Stiffness (kPa)	MN1	30.28 (28.84–34.02)	30.70 (28.63–33.18)	27.81 (27.43–31.08)	0.319^a^
	MN2	21.54 (19.87–24.21)	22.36 (20.43–24.15)	21.10 (19.61–25.31)	0.839^a^
	MN3	21.58 ± 2.43	22.00 ± 2.53	22.35 ± 2.78	0.650^b^
Viscosity (Pa·s)	MN1	1.84 ± 0.61	1.90 ± 0.77	1.39 ± 0.43	0.168^b^
	MN2	1.80 (1.54–3.89)	1.95 (1.67–2.15)	2.05 (1.86–2.71)	0.393^a^
	MN3	1.69 (1.34–2.93)	1.66 (1.42–2.32)	2.49 (1.27–2.53)	0.555^a^

Abbreviations: MN1, median nerve at the carpal tunnel; MN2, median nerve at mid-forearm; MN3, median nerve at 5 cm proximal to the elbow joint; Pa·s, pascal seconds; ^a^ Kruskal–Wallis H test, Median (Q25-Q75); ^b^ one-way analysis of variance (ANOVA)

BMI-based subgroup analysis also showed no significant differences in stiffness or viscosity across the three BMI categories (< 18.5, 18.5–23.9, ≥ 24 kg/m²) (all *p* > 0.05; Table 6). At MN1, stiffness values were 31.56 kPa (29.91–34.43), 29.88 kPa (28.31–32.96), and 30.65 kPa (28.04–34.08) across the underweight, normal weight, and overweight/obese groups, respectively (*p* = 0.253). Corresponding viscosity values were 1.74 ± 0.88 Pa·s, 1.74 ± 0.66 Pa·s, and 1.97 ± 0.50 Pa·s (*p* = 0.240). Similar nonsignificant findings were observed at MN2 and MN3.

**Table 6:** Comparisons of stiffness and viscosity values of the MN on the dominant side in volunteers according to BMI group (*n* = 98)

Parameter	Site	G1 (*n* = 7)	G2 (*n* = 58)	G3 (*n* = 33)	*p*-value
Stiffness (kPa)	MN1	31.56 (29.91–34.43)	29.88 (28.31–32.96)	30.65 (28.04–34.08)	0.253^a^
	MN2	21.54 (20.17–28.52)	21.30 (19.70-24.33)	21.97 (20.40- 23.84)	0.618^a^
	MN3	23.63 ± 2.91	21.35 ± 2.17	21.89 ± 2.68	0.056^b^
Viscosity (Pa·s)	MN1	1.74 ± 0.88	1.74 ± 0.66	1.97 ± 0.50	0.240^b^
	MN2	1.51 (0.89–2.21)	1.61 (1.07–1.86)	1.57 (1.27–1.83)	0.815^a^
	MN3	1.48 (1.00-2.17)	1.34 (1.05–1.61)	1.40 (1.16–1.84)	0.528^a^

Abbreviations: BMI, body mass index; MN1, median nerve at the carpal tunnel; MN2, median nerve at mid-forearm; MN3, median nerve at 5 cm proximal to the elbow joint; G1, group 1 (BMI < 18.5); G2, group 2 (18.5 ~ < 24); G3, group 3 (BMI ≥ 24); Pa.s, pascal seconds; ^a^ Kruskal–Wallis H test, Median (Q25-Q75); ^b^ one-way analysis of variance (ANOVA)

## Discussion

Ultrasound viscoelastic techniques, including SWE and shear wave dispersion imaging, have emerged as valuable tools for characterizing the biomechanical properties of biological tissues [5, 10]. While these approaches have been widely applied to hepatic and musculoskeletal systems [14–16], their application to peripheral nerves remains limited. The present study is among the first to establish normative viscosity and stiffness values of the MN in healthy adults and to explore demographic influences, thereby providing a foundation for future diagnostic applications.

Symmetry of viscosity and stiffness between left and right MNs was confirmed, supporting the feasibility of using the contralateral limb as an internal reference in unilateral neuropathies [17]. However, in systemic or bilateral neuropathies such as diabetic polyneuropathy, population-based normative values remain indispensable for distinguishing pathological deviations.

Our findings revealed that both viscosity and stiffness of the MN varied significantly with anatomical location, with the highest values consistently observed at the MN1. This location-specific variation may be attributed to the biomechanical environment surrounding MN1 [18], including the presence of rigid anatomical structures such as the transverse carpal ligament, flexor tendons, and adjacent bones [19], which could influence shear wave propagation. In addition, the superficial positioning of the MN at MN1 may increase its sensitivity to subtle probe pressure effects despite standardized scanning protocols. Importantly, our reproducibility analysis confirmed high intra- and inter-operator agreement, with intra-operator ICCs ranging from 0.78 to 0.91 (good–excellent) and inter-operator ICCs ranging from 0.66 to 0.75 (moderate–good), thereby supporting the reliability of anatomical landmarks in ensuring consistent STVi measurements.

Gender-based differences were also observed, with males exhibiting significantly higher viscosity and stiffness values at MN1 and MN2, even after accounting for CSA differences. This suggests that the observed sex differences are likely attributable to intrinsic biological factors, such as androgen-mediated [20] connective tissue remodeling or differences in muscle mass and biomechanical loading. Conversely, increased subcutaneous fat in females could contribute to partial shear wave attenuation [21, 22]. These findings highlight the necessity of establishing sex-specific normative ranges and diagnostic thresholds in future clinical applications, particularly when interpreting abnormal STVi values in suspected neuropathies.

In contrast to sex and location, no significant age-related effects were observed in our cohort. These results are consistent with previous studies reporting no association between peripheral nerve stiffness and age [19, 23, 24]. Although a prior version of our analysis suggested reduced MN1 viscosity in older adults, this effect was not maintained after incorporating reproducibility and subgroup validations. Given the limited sample size of participants aged ≥ 60 years, the absence of an age-related trend should be interpreted with caution. Larger age-stratified studies will be required to confirm this observation.

BMI showed no significant correlation with either viscosity or stiffness at any anatomical location, which is consistent with prior observations [25–27]. This suggests that within a moderate range of body composition, peripheral nerve viscosity and stiffness remain relatively stable. Nevertheless, the small number of obese participants in our study limits extrapolation, and future investigations should evaluate the impact of extreme BMI values.

Several limitations should be acknowledged. First, although clinical and sonographic evaluations were rigorously applied to exclude peripheral neuropathy, the lack of EMG the diagnostic gold standard raises the possibility of undetected subclinical abnormalities. Second, the study did not compare STVi-derived parameters with established gold standards such as EMG or histopathology, which will be critical for validating clinical diagnostic significance. Third, the relatively small number of elderly and obese participants may have reduced statistical power to detect demo establishing normative values in healthy adults’ graphic associations. Fourth, reproducibility analyses were conducted in a subset of 20 participants; while ICC values indicated good reliability, broader validation across multi-operator and multi-center settings will be required. Finally, the exclusion of patients with confirmed neuropathies restricts direct clinical translation, but establishing normative values in healthy adults is a necessary prerequisite for defining pathological thresholds in future research.

Taken together, this study provides the first normative reference values for viscosity and stiffness of the median nerve in healthy adults using STVi, demonstrating both the feasibility and reproducibility of this novel technique. Importantly, these normative data establish a necessary baseline for clinical translation: (i) in unilateral neuropathies such as carpal tunnel syndrome, the confirmed left–right symmetry supports the use of the contralateral limb as an internal reference; (ii) in systemic or bilateral neuropathies such as diabetic polyneuropathy, population-based reference values are indispensable for distinguishing pathological deviations; and (iii) the demonstrated reproducibility and demographic influences lay the groundwork for integrating STVi into future patient cohorts. Although further validation against gold standards such as EMG and expansion to multi-center studies are required, our findings provide a methodological and clinical basis for applying STVi in the early diagnosis and longitudinal monitoring of peripheral neuropathies.

## Conclusions

This study demonstrated that STVi is a feasible and reliable technique for quantifying the viscoelastic properties of the median nerve in healthy adults. Significant effects of anatomical location and sex were observed, whereas age and BMI showed no detectable influence. By providing normative reference values, our work lays the groundwork for future large-scale, longitudinal, and disease-specific studies to validate the diagnostic and prognostic potential of STVi in peripheral neuropathies.

## Supplementary Information

Below is the link to the electronic supplementary material.

Supplementary Material 1

## Data Availability

All data generated or analyzed during this study are included in this published article.

## Abbreviations

MN: Median Nerve
STVi: Sound Touch Viscosity
SWE: Shear Wave Elastography
BMI: Body Mass Index
Pa·s: Pascal Seconds
EMG: Electromyography
CSA: Cross-sectional Area
ROI: Region of Interest
M-STB index: Motion Stability Index
RLB: Reliability
SD: Standard Deviation
ANOVA: Analysis of Variance
ICC: Intra-class Correlation Coefficient
